# Light-sensitive Ca^2+^ signaling in the mammalian choroid

**DOI:** 10.1073/pnas.2418429121

**Published:** 2024-11-08

**Authors:** Ahmed M. Eltanahy, Alex Aupetit, Ethan D. Buhr, Russell N. Van Gelder, Albert L. Gonzales

**Affiliations:** ^a^Department of Physiology and Cell Biology, Center for Molecular and Cellular Signaling in the Cardiovascular System, University of Nevada, Reno School of Medicine, Reno, NV 89557-0318; ^b^Department of Ophthalmology, University of Washington, Seattle, WA 98104; ^c^Roger and Angie Karalis Retina Center, Department of Ophthalmology, University of Washington, Seattle, WA 98104; ^d^Department of Laboratory Medicine and Pathology, University of Washington, Seattle, WA 98104; ^e^Department of Neurobiology & Biophysics, University of Washington, Seattle, WA 98104

**Keywords:** Ca^2+^ signaling, opsins, vasoconstriction, fluid homeostasis

## Abstract

The choroid is the thin, vasculature-filled layer of the eye situated between the sclera and the retina, where it serves the metabolic needs of the light-sensing photoreceptors in the retina. Illumination of the interior surface of the back of the eye (fundus) is a critical regulator of subretinal fluid homeostasis, which determines the overall shape of the eye, but it is also important for choroidal perfusion. Noted for having some of the highest blood flow rates in the body, the choroidal vasculature has been reported to lack intrinsic, intravascular pressure-induced (myogenic) autoregulatory mechanisms. Here, we ask how light directly regulates choroid perfusion and ocular fluid homeostasis, testing the hypothesis that light facilitates ocular fluid absorption by directly increasing choroid endothelial permeability and decreasing choroid perfusion. Utilizing ex vivo pressurized whole-choroid and whole-eye preparations from mice expressing cell-specific Ca^2+^ indicators, we found that the choroidal vasculature has two intrinsically light-sensitive Ca^2+^-signaling mechanisms: One increases Ca^2+^-dependent production of nitric oxide in choroidal endothelial cells; the other promotes vasoconstriction through Ca^2+^ elevation in vascular smooth muscle cells. In addition, we found that choroidal flow, or pressure, modulates endothelial and smooth muscle photosensitivity and trans-retinal absorption of fluid into the choroid. These results collectively suggest that the choroid vasculature exhibits an inverted form of autoregulatory control, where pressure- and light-induced mechanisms work in opposition to regulate blood flow and maintain fluid balance in response to changes in light and dark, aligning with the metabolic needs of photoreceptors.

## Significance

The choroid vasculature, which exhibits the highest blood flow per unit weight compared to any other human tissue, functions to meet the metabolic needs of photoreceptors in the retina and maintain the fluid balance and overall shape of the eye. Changes in the state of the choroid have significant implications for the development and progression of myopia, a condition estimated to affect half the global population by 2050. Here, we demonstrate that the choroid vasculature, including endothelial and mural cells, possesses the machinery to directly sense light, enabling them to regulate vascular tone, deliver oxygen and nutrients, and efficiently remove waste.

The choroid, situated in the posterior eye between the sclera and the retinal pigment epithelium (RPE), is home to a complex vascular network that serves to support the health of photoreceptor cells in the outer retina (1). In addition to receiving approximately 85% of the blood entering the eye (2, 3), the choroid circulation boasts some of the highest blood flow rates in the body (3)—nearly 10-times greater than that in the cerebral or retinal circulation (4). These high-flow conditions result in rapid red blood cell (RBC) transit times that limit arteriovenous oxygen (O_2_) extraction rates to only 2 to 4% (5–8) versus the 35 to 38% observed in the retinal circulation (5, 9). This creates a unique scenario in which a reduction in choroidal blood flow would be required to increase O_2_ levels in the tissue (10, 11).

In addition to its traditional blood-delivery role, the choroidal circulation regulates choroid tissue thickness, intraocular pressure, and the overall fluid balance of the eye (12–14). These functions impact the shape of the eye and how light is focused onto the retina. With the growing prevalence of myopia, or nearsightedness—estimates suggest that almost half the global population will suffer from myopia by 2050 (15)—it has become increasingly important to understand the fundamental mechanisms that regulate choroidal blood flow.

Intravascular pressure-induced constriction, a process intrinsic to smooth muscle termed the myogenic response, is a major autoregulatory mechanism that confers the ability of blood vessels to maintain blood flow despite changes in intravascular pressure. It was initially reported that the choroid vasculature lacks intrinsic autoregulatory mechanisms (10), a finding that arguably made sense given the presumptive adaptations of the choroid vascular to extremely high blood flow rates, which would appear to obviate the need for a mechanism to limit blood flow, even under high intraluminal pressures. However, recent studies have reported that changes in intraocular or mean arterial pressure can alter choroidal blood flow (14, 16, 17). Several reports have also alluded to the presence of a light-sensitive mechanism that affects choroidal blood flow (10, 11, 18–20), but these studies have focused on photoreceptors or neuronal innervation (11, 20) as the primary mechanisms involved in linking light to vascular function. An alternative frame of reference is provided by a study by Longden et al. (21), who showed that brain capillary networks constitute a “sensory web” that is capable of detecting and communicating the metabolic state of neuronal tissue and responding by directing blood flow to regions in metabolic need. Here, we hypothesize that the choroid vasculature possesses an analogous intrinsic apparatus that contributes to a type of “autoregulation” of choroidal blood flow, but one that is light-sensitive and may be critical for light/dark adaptation processes.

Vision, directly and indirectly, depends on opsins, a family of light-sensitive G protein-coupled receptors (GPCRs). The human genome contains eight opsins, including rhodopsin (RHO), three cone opsins (OPN1LW, OPN1MW, and OPN1SW), encephalopsin (OPN3), melanopsin (OPN4), peropsin (RRH), and neuropsin (OPN5) (22). While rhodopsin and the three cone opsins are primarily involved in image formation, other members of the opsin family play crucial roles in non-image-forming functions, including circadian photoentrainment, wound healing, vascular development, and regulation of metabolism (23–30). Similar to other GPCRs, light-activated opsins transmit intracellular signals via Gα subunits, which generate secondary messengers that regulate a number of intracellular functions, including intracellular Ca^2+^ homeostasis.

We propose that choroidal vascular cells possess opsin-mediated, light-sensitive machinery that regulates choroidal hemodynamics and subretinal fluid homeostasis. We tested this proposition by studying the intrinsic light sensitivity of mammalian choroid vascular cells using a physiological, perfused ex vivo choroid and whole-eye preparations (31) from transgenic mice expressing the genetically encoded Ca^2+^ sensor, GCaMP6f expressed specifically in endothelial and mural cells. In addition, we studied expression of the nonvisual opsins, *Opn3*, *Opn4,* and *Opn5* in the choroid circulation using transgenic reporter mice.

## Results

### Violet Light Stimulates Cell-Specific Ca^2+^ Signals in the Choriocapillaris.

In the choroid, large arterioles diverge to form the central choriocapillaris, which is arranged in an interconnected honeycomb configuration that decreases blood flow resistance and provides a large surface area for exchange between the choroid and RPE (32). Several reports have demonstrated light-dependent changes in choroidal blood flow (10, 11, 16, 18–20, 33); however, the relevant photosensitive machinery has yet to be elucidated. As a key second messenger and regulator of vasculature function, Ca^2+^ dynamics in endothelial cells and mural cells of the choroid could be key targets of light, which could act through opsins to stimulate changes in the contractile state of choroid vessels. Using an en face choroidal tissue preparation, a custom-built ex vivo perfusion chamber, and transgenic mice with cell-specific expression of the genetically encoded Ca^2+^ sensor, GCaMP6f, we examined the effects of light on choroidal endothelial and mural cell Ca^2+^ dynamics and vascular diameter.

**Fig. 1.**
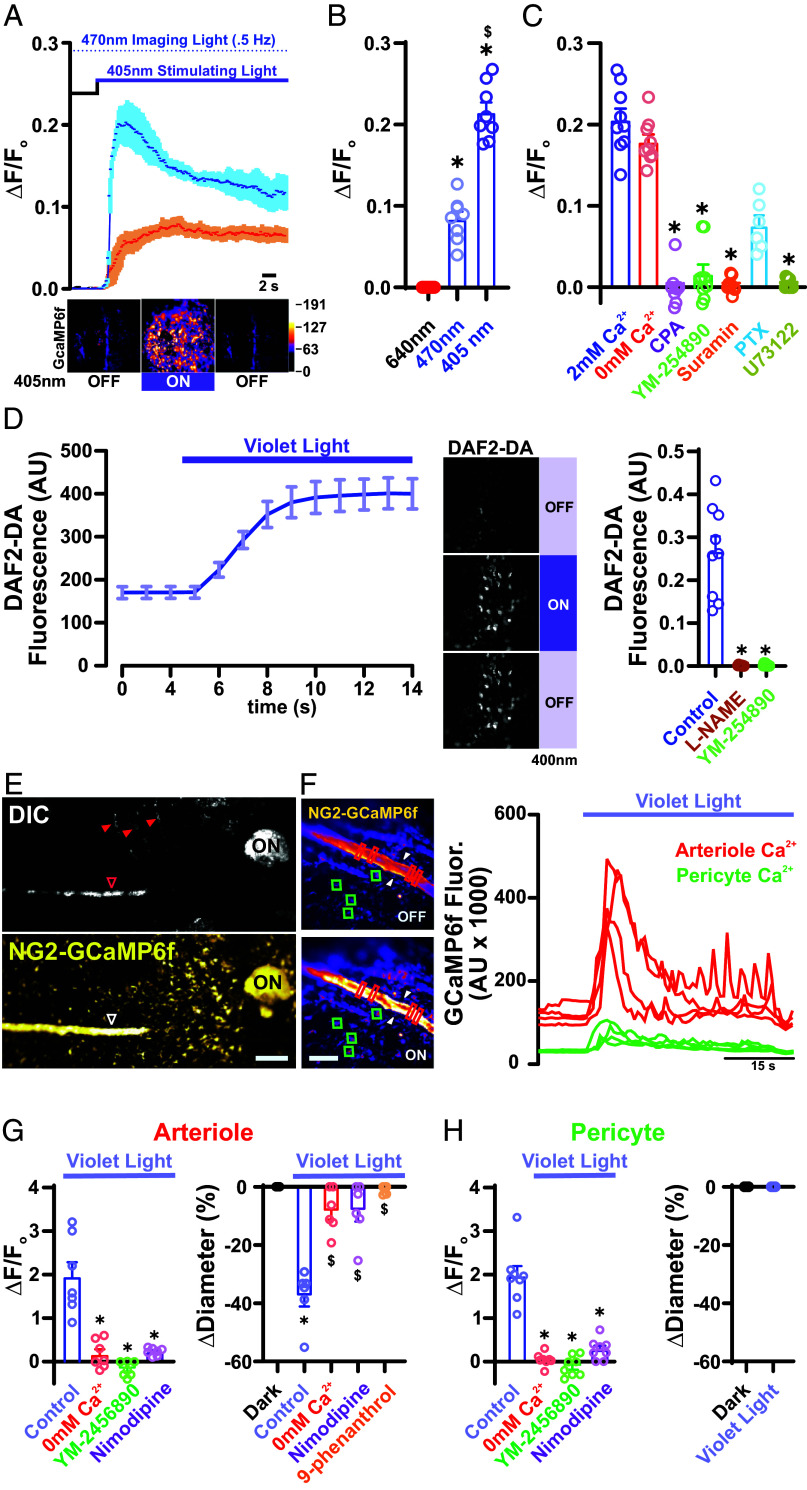
Violet light stimulate cell-specific Ca^2+^ signals in the choriocapillaris. (*A*) *Top*: Representative trace of averaged GcAMP6f fluorescence in the choriocapillaris endothelium in response to constant violet light stimulation (405 nm) at 2.04 × 10^14^ photons/cm^2^/s (orange) and 6.1 × 10^14^ photons/cm^2^/s (blue) (n = 3 choroid preparations from 3 mice per group). (*A*) *Bottom*: Representative micrographs of a choroidal preparation from a Cdh5-GCaMP6f mouse showing Ca^2+^ levels before (*Left*), 5 s after (*Middle*), and 10 s after (*Right*) light stimulation. (*B*) Summary data showing changes in GcaMP6f fluorescence (∆F/F_o_) in the choriocapillaris endothelium in response to light stimulation (6.1 × 10^14^ photons/cm^2^/s) at 405, 470, and 640 nm. Data are presented as means ± SEM [error bars; *P < 0.05; n = 5 regions of interest (ROIs) in 5 choroids from 3 mice per group]. (*C*) Summary data showing changes in fluorescence in the choriocapillaris endothelium in response to light stimulation (405 nm, 6.1 × 10^14^ photons/cm^2^/s) under control conditions (2 mM Ca^2+^) and with 0 mM Ca^2+^ in the absence or presence of CPA (5 µmol/L), YM-254890 (1 µmol/L), suramin (5 μM), or U73122 (3 μM). Data are presented as means ± SEM (error bars; *P < 0.05; n = 9 ROIs in 5 choroids from 5 mice per group). (*D*, *Left*) Traces showing averaged fluorescence of the NO indicator, DAF2-DA (5 μM), in the choriocapillaris endothelium in response to violet light stimulation (405 nm, 6.1 × 10^14^ photons/cm^2^/s). (*D*, *Middle*) Representative micrographs of a choroidal preparation showing DAF2-DA fluorescence before (*Top*), 5 s after (*Middle*), and 10 s after (*Bottom*) light stimulation. (Scale bars, 20 μm.) (*D*, *Right*) Summary data showing changes in DAF2-DA fluorescence in the choriocapillaris endothelium in response to violet light stimulation (405 nm, 6.1 × 10^14^ photons/cm^2^/s) under control conditions and in the presence of L-NAME (10 μM) or YM-254890 (1 μM). Data are presented as means ± SEM (error bars; *P < 0.05; n = 9 ROIs in 6 choroids from 3 mice per group). (*E*, *Top*) Representative low-magnification contrast light image of the en face whole mount choroid preparation from an NG2-GCaMP6f mouse showing the hypopigmented areas of choroidal fissures where vessels and nerves run (arrowheads). (*E*, *Bottom*) Fluorescence micrograph of the same region shown in the DIC image above identifying arterioles (open arrowhead) and pericytes. (Scale bars, 300 µm.) (*F*, *Left*) Representative images of a region from an NG2-GcaMP6f mouse showing an arteriole and capillary pericytes, highlighted by red rectangular and green square ROIs, respectively, under light-off conditions (*Top*) and following stimulation with violet light (405 nm, 6.1 × 10^14^ photons/cm^2^/s) (*Bottom*). (*F*, *Right*) Traces showing Ca^2+^ levels in ROIs in the arteriole (red rectangles) and pericytes (green squares) in *B* (*Bottom*). (Scale bar, 10 µm.) (*G*, *Left*) Summary data showing changes in GCaMP6f fluorescence in arterioles in response to violet stimulation (405 nm, 6.1 × 10^14^ photons/cm^2^/s) under control conditions (2 mM Ca^2+^) and with 0 mM Ca^2+^ in the absence or presence of YM-254890 (1 μM) or nimodipine (5 μM). Data are presented as means ± SEM (error bars; *P < 0.05; n = 8 ROIs in 6 choroids from 3 mice per group). (*G*, *Right*) Summary data showing changes in ciliary arteriolar diameter in response to violet light stimulation (405 nm, 6.1 × 10^14^ photons/cm^2^/s) under control conditions (2 mM Ca^2+^) and with 0 mM Ca^2+^ in the absence or presence of YM-254890 (1 µmol/L), nimodipine (5 μM), or 9-phenanthrol (30 μM). Data are presented as means ± SEM (error bars; *P < 0.05; n = 6 vessels in 6 choroids from 3 mice per group). (*H*, *Left*) Summary data showing changes in GCaMP6f fluorescence in choriocapillaris pericytes in response to violet light stimulation (405 nm, 6.1 × 10^14^ photons/cm^2^/s) under control conditions (2 mM Ca^2+^) and with 0 mM Ca^2+^ in the absence and presence of YM-254890 (1 μM) or nimodipine (5 μM). (*H*, *Right*) Summary data showing changes in capillary vessels in response to violet light stimulation (405 nm, 6.1 × 10^14^ photons/cm^2^/s) under control conditions (2 mM Ca^2+^). Data are presented as means ± SEM (error bars; *P < 0.05; n = 7 ROIs in 6 choroids from 3 mice per group).

To examine Ca^2+^ dynamics in choroid endothelial cells, we used *Cdh5*-GCaMP6f transgenic mice, in which the GFP-based Ca^2+^ indicator, GCaMP6f, is specifically expressed in endothelial cells under the control of the cadherin-5 (*Cdh5*) promoter. To simultaneously image and photostimulate the choroid tissue, we used two multimodal solid-state laser diode illuminators (LDI), one connected to a confocal/widefield microscope for imaging, and the second connected to a pattern illuminator for photostimulation. With this configuration, we could independently control the temporal and spatial delivery of various wavelengths of light for imaging and photostimulation. The peak excitation (λ_ex_) and emissions (λ_em_) wavelengths of GCaMP6f are 496 nm and 513 nm, respectively. To minimize potential cross-stimulation by our imaging light, we imaged at a rate of 0.5 Hz and a power less than 2.36 × 10^14^ photons/cm^2^/s. Flat-mounted choroids isolated from transgenic *Cdh5*-GCaMP6f mice were perfused with a physiological saline solution containing 6 μM 9-cis retinal for 1 h before light exposure. Under this configuration, we observed a direct photic response to violet light stimulation (405 nm, 2.04 × 10^14^ photons/cm^2^/s and 6.1 × 10^14^ photons/cm^2^/s) that manifested as an increase in intracellular Ca^2+^ levels in choriocapillaris endothelial cells (Fig. 1*A*). Ca^2+^ responses to blue light (470 nm, 4.5 × 10^14^ photons/cm^2^/s) were significantly diminished and were absent following stimulation with red light (640 nm, 9.52 × 10^14^ photons/cm^2^/s) (Fig. 1*B*). To examine the potential for cross-stimulation by the wavelength of light used to image GCaMP6f (470 nm), we loaded choroids with Cabryte 630, a red-shifted Ca^2+^ indicator (λ_ex_ = 607 and λ_em_ = 624). Under these conditions, multiple unidentified cells responded to illumination with 405 nm light and, to a lesser extent, to 470 nm light (*SI Appendix*, Fig. S1). Last, we found that pretreating choroids with tetrodotoxin (TTX; 500 nM), a voltage-gated sodium (Na_V_) channel inhibitor that abolishes neuronal activity (*SI Appendix*, Fig. S1), failed to prevent 405 nm light-induced increases in Ca^2+^ in choroid endothelial cells; similar effects were observed with saturating concentrations of TEMPOL (10 μM), a scavenger of reactive oxygen species (ROS) (*SI Appendix*, Fig. S1). These observations suggest that the light response is independent of neuronal activity and laser-induced ROS generation. To assess the contributions of extracellular versus intracellular Ca^2+^ to this signaling pathway, we stimulated choroid endothelial cells with violet light under Ca^2+^-free extracellular conditions (5 to 10 min) or in the presence of the sarco/endoplasmic reticulum Ca^2+^-ATPase (SERCA) inhibitor, cyclopiazonic acid (CPA). We found no significant difference in Ca^2+^ response compared with that in normal 2 mM extracellular Ca^2+^ (Fig. 1*C*), whereas administration of CPA (5 μM) blocked the response (Fig. 1*C*), suggesting that violet light stimulation leads to the release of Ca^2+^ from internal stores. To examine the role of G_q_-type GPCRs (G_q_PCRs) and their intracellular target, phospholipase C (PLC), we used YM-254890 (1 μM), a cyclic depsipeptide that potently inhibits G_q_PCR signaling by preventing guanosine diphosphate release from the G_q_α subunit; suramin (10 μM), a broad-spectrum inhibitor of Gα and Gβγ subunit association (34); pertussis toxin (200 ng/mL, 4 h), a blocker of heterotrimeric (G_αβγ_) G protein–receptor interactions; and U73122 (10 μM*)*, an inhibitor of PLC. In each case, these inhibitors blunted or eliminated the Ca^2+^ response (Fig. 1*C*), suggesting that violet light stimulates a G_q_PCR-dependent activation of PLC, leading to the release of Ca^2+^ from internal stores.

Nitric oxide (NO), synthesized from L-arginine by Ca^2+^/ calmodulin-dependent NO synthase (NOS) in vascular endothelial cells, is a potent vasodilator. To examine whether violet light-induced increases in intracellular Ca^2+^ lead to an increase in NO in choroid capillary endothelial cells, we used the NO fluorescence indicator, DAF-2DA (4-amino-5-methylamino-2′,7′difluorescein). We first tested the sensitivity of DAF-2DA for detection of intracellular nitrosation by obtaining time-lapsed images of DAF-2T–loaded, flat-mounted choroids every 2 min after illumination with violet light (6.1 × 10^14^ photons/cm^2^/s). Violet light stimulation induced an immediate increase in the DAF-2T fluorescence signal that was diffusely distributed throughout the cell (Fig. 1*D*). This suggested that DAF-2T does not errantly concentrate in specific organelles, making it an appropriate tool for detecting diffusible NO derived from eNOS in the choroid. We further found that the NOS inhibitor, L-NAME (N-omega-nitro-L-arginine methyl ester hydrochloride), prevented the violet light-induced increase in DAF-2T signaling. Last, inhibition of G_q_PCRs with YM-254890 (10 μM) completely inhibited violet light-stimulation–induced release of NO by choriocapillaris endothelial cells (Fig. 1*D*). Taken together, these observations indicate that the choriocapillaris endothelium is intrinsically sensitive to a violet light-stimulated, G_q_PCR-dependent Ca^2+^–NO signaling pathway. In addition, these data suggest that choriocapillaris endothelial cells are capable of directly sensing and responding to the light/dark environment of the choroid.

The contractile state of feeding arterioles regulates the bulk flow of blood entering a capillary network. Previous work regarding whether flickering light affects choroid blood flow/volume has been contentious (10, 11, 35). However, whether the choroid is intrinsically sensitive to light or the observed effect is instead part of a bystander effect of retinal activity has remained unclear. Therefore, we sought to determine whether mammalian choroid mural cells—vascular smooth muscle cells (vSMCs) and capillary pericytes—are intrinsically sensitive to light by testing whether violet light was capable of stimulating an increase in Ca^2+^ in arteriole SMCs and/or capillary pericytes. To measure changes in Ca^2+^ levels specifically in choroid SMCs and pericytes, we used isolated choroidal preparations obtained from *NG2*-GCaMP6f transgenic mice expressing GCaMP6f specifically in mural cells. An examination of the choroid by differential interference contrast (DIC) microscopy showed regions of thinned or incomplete coverage by choroidal melanin, traditionally considered tracks for ciliary vessels and nerves (36), that might be important for the efficiency of light-sensing by choroidal arterioles (Fig. 1*E*). We found that violet light stimulation (6.1 × 10^14^ photons/cm^2^/s) induced a robust increase in intracellular Ca^2+^ in arteriolar SMCs and pericytes in the choroid vasculature from *NG2*-GCaMP6f transgenic mice (Fig. 1*F*). Previous reports (37) have suggested that other nonvascular cell types within the choroid might also express *Cspg4.* Using choroids isolated from *Myh11-*GCaMP6f transgenic mice, in which the GFP-based Ca^2+^ indicator, GcaMP6f, is specifically expressed in mural cells under the control of the myosin regulatory light chain 11 (*myh11*) promoter we observed increases in mural cells Ca^2+^ following similar light stimulation (*SI Appendix*, Fig. S2). In contrast to our findings in capillary endothelial cells, the light response in mural cells was diminished in Ca^2+^-free extracellular buffer (Fig. 1 *G* and *H*, *Left*), suggesting that violet light leads to an influx of Ca^2+^ from the extracellular space. Preincubation with the specific G_q/11_ inhibitor, YM-254890 (1 μM), or nimodipine (10 μM), a voltage-gated Ca^2+^ channel (VGCC) blocker, inhibited violet light-induced mural cell Ca^2+^ signaling (Fig. 1 *G* and *H*, *Left*). Consistent with an increase in Ca^2+^, violet light stimulation led to constriction of ciliary arteries and arterioles, but interestingly did not affect the contractile state of pericyte-enwrapped choriocapillaris vessels (Fig. 1 *G* and *H*, *Right*). In addition, treatment with 9-phenanthrol (20 μM), which inhibits Ca^2+^-dependent membrane depolarization mediated by the nonselective cation channel TRPM4 (transient receptor potential melastatin-4) and Ca^2+^-activated chloride channel ANO1 (anoctamin 1) (38), prevented the vasoconstriction to violet light, suggesting that a TRPM4/ANO1-dependent depolarizing current is activated by violet light stimulation. Among TRP channels, Ca^2+^ dependence is unique to TRPM4 and TRPM5 (39), suggesting that violet light-mediated increases in intracellular Ca^2+^ may be an important regulatory stimulus under physiological conditions. These data suggest that violet light has two cellular effects: It increases intracellular Ca^2+^ and NO production in choroid capillary endothelial cells, and it increases intracellular SMC and pericyte Ca^2+^, causing constriction of choroid arterioles.

### Intrinsic Light Sensitivity of the Nonhuman Primate Choroid.

Although a powerful genetic model, mice are nocturnal animals and therefore are active primarily in dark or low-light conditions. Unlike rodents, both humans and some nonhuman primates are diurnal and rely primarily on visual cues to extract information from their social environments. Therefore, we asked whether violet light stimulated a similar response in the monkey choroid. To address this, we used freshly enucleated eyes from 2-year-old cynomolgus macaques (*Macaca fascicularis*). Using the membrane-permeant Ca^2+^-sensitive dye, Fluo4-AM, we found that violet light induced an immediate increase in choriocapillaris endothelial Ca^2+^ in isolated ex vivo monkey choriocapillaris patches (*SI Appendix*, Fig. S3). This response was blocked by the G_q_PCR inhibitor, YM254890 (1 μM, *SI Appendix*, Fig. S3). We further found that violet light-induced constriction of ciliary arterioles that feed the choroid vasculature. These data suggest that diurnal and nocturnal animals both possess an intrinsic light-sensitive machinery in the choriocapillaris that is critical for ocular physiology and light/dark adaptation processes.

### Autoregulatory Control of Choriocapillaris Endothelial Photosensitivity.

**Fig. 2.**
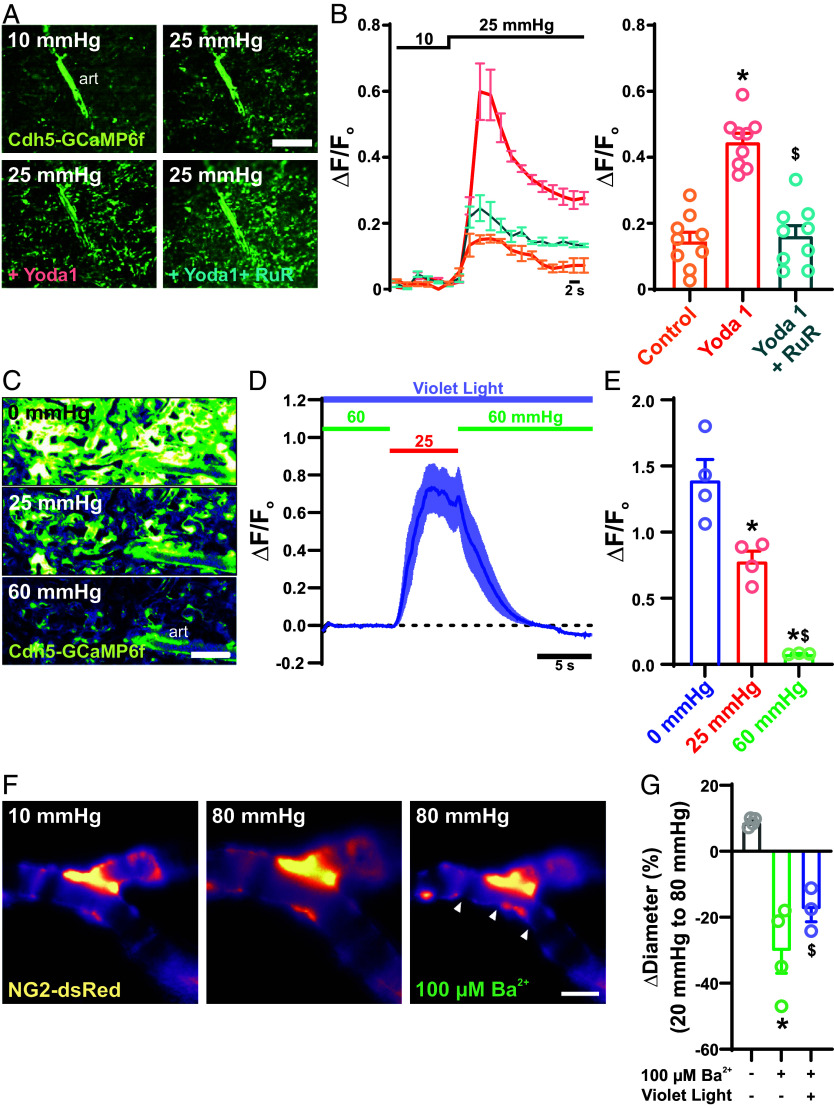
Intraluminal flow and pressure regulate light-dependent signaling. (*A*) Representative images of a choroidal preparation from a Cdh5-GCaMP6f mouse showing Ca^2+^ fluorescence in the choriocapillaris endothelium in response to increases in intraluminal pressure from 10 mmHg to 25 mmHg under control conditions and in the presence of bath-applied Yoda 1 (10 μM) or Yoda 1 + ruthenium red (30 μM) (n = 3 choroid preparations from 3 mice). (*B*) Traces (*Left*) and summary data (*Right*) showing averaged fluorescence of Ca^2+^ transients in the choriocapillaris endothelium in response application of Yoda 1 (10 μM) (error bars; *P < 0.05 to control and ^$^*P* < 0.05 to yoda1. n = 3 choroid preparations from 3 mice per group). (Scale bars, 300 μm.) (*C*–*E*) Representative images (*C*), traces (*D*), and summary data (*E*) of Ca^2+^ transients in the choriocapillaris endothelium in response to violet light stimulation (405 nm, 6.1 × 10^14^ photons/cm^2^/s) at 0, 20, and 60 mmHg (error bars; *P < 0.05 to 0 mmHg and ^$^*P* < 0.05 to 25 mmHg, n = 3 choroid preparations from 3 mice). (*F* and *G*) Representative images (*F*) and summary data (*G*) showing pressure-induced arteriole constriction in the absence of light stimulation, without and with the Kir channel blocker Ba^2+^ (100 µM), and with violet light stimulation (6.1 × 10^14^ photons/cm^2^/s) in the presence of Ba^2+^ (error bars; *P < 0.05 to control and ^$^*P* < 0.05 to 100 µM Ba^2+^, n = 6 ROIs in 3 choroids from 3 mice per group).

Blood vessels have the intrinsic capacity to sense and dynamically respond to changes in intraluminal pressure through autoregulatory mechanisms, prominently including the myogenic response. Therefore, we were interested in determining how luminal forces might affect light-sensitive signaling processes within the choroid. To control intraluminal perfusion and examine its effects, we developed an ex vivo pressurized choroid preparation, modified from our pressurized whole-eye model (31), that provides the experimental advantage of allowing imaging access to the choroid vasculature. In this ex vivo pressurized choroid preparation, the choroid is pinned down en face with the retinal and choroid surface facing up, and then the ophthalmic artery is cannulated and pressurized. Pressure and flow changes at the ophthalmic artery have been shown to translate to changes in pressure at the microvascular level within the retina (40, 41). To examine whether flow stimulates choroid capillary endothelial Ca^2+^ activity, we monitored changes in Ca^2+^ levels specifically in the choroid endothelium using *Cdh5*-GCaMP6f transgenic mice. To test the validity of the preparation, we measured changes in endothelial Ca^2+^ following elevation of pressure at the ophthalmic artery from 10 to 25 mmHg. This increase in pressure, and thus the resulting increase in luminal flow, led to an immediate increase in choroid capillary endothelial Ca^2+^ (Fig. 2 *A* and *B*). PIEZO1, a mechanically activated, Ca^2+^-permeable ion channel, has been proposed to act as a flow sensor in capillary endothelial cells (40). Consistent with this proposed role, activation of PIEZO1 channels with the selective agonist, Yoda1 (30 μM) (40), potentiated pressure/flow-induced Ca^2+^ transients in choroid capillary endothelial cells. This effect was blocked by ruthenium red (10 μM), a broad spectrum ion channel inhibitor that effectively blocks PIEZO channels (42) (Fig. 2*B*). These data suggest that, as is the case in other vascular beds (40, 43), shear stress stimulates PIEZO1 channels, eliciting endothelial cell Ca^2+^ transients in the choriocapillaris.

Increases in cytosolic Ca^2+^ are key to the production of NO in endothelial cells. Our data (above) show that violet light and intraluminal flow can both independently increase intracellular Ca^2+^ in choroidal capillary endothelial cells. Therefore, we sought to determine whether, and how, these mechanisms of Ca^2+^ mobilization interact with each other. Interestingly, we found that violet light-induced choroid capillary endothelial cell Ca^2+^ signaling was negatively correlated with intraluminal pressure such that increases in capillary pressure (from 0 to 25 and 60 mmHg) reversibly decreased violet light-induced responses (Fig. 2 *C*–*E*). These data suggest that violet light and intraluminal flow function in opposition to each other, with increases in intraluminal pressure/flow through the choroid capillaries decreasing the cell’s sensitivity to violet light; thus, the choriocapillaris is maximally light-sensitive under low-flow conditions.

vSMCs are intrinsically capable of sensing and constricting to changes in intraluminal pressures, a process termed the myogenic response that serves to maintain relatively constant blood flow in the face of changing blood pressure. The presence of this vascular autoregulatory mechanism in the choroid vasculature (44) has been debated. To directly test for the presence of a myogenic mechanism, we investigated changes in arterial diameter in response to pressure in our ex vivo pressurized choroid vasculature preparation. Increasing intraluminal pressure at the ophthalmic artery from 20 to 80 mmHg did not cause constriction of choroid arterioles, but instead induced a slight distention of the vessels (Fig. 2*F*). Although myogenic responses in different vascular beds may differ (45), these data seemingly suggest that choroid arterioles lack this autoregulatory myogenic mechanism. A similar phenomenon was reported in the bladder vasculature, where it was ultimately determined that the apparent absence of a myogenic response reflected a hyperpolarized membrane potential, attributable to robust activity of the strong inwardly rectifying K^+^ (K_ir_) channel, that resisted the depolarizing influence of pressure (45). Consistent with these observations, we found that inhibition of K_ir_ channels with the selective pore-blocker, Ba^2+^ (100 µM), led to restoration of pressure-induced constriction of choroid arterioles (Fig. 2 *F* and *G*). This suggests that, despite the presence of pressure-sensing machinery and vascular autoregulatory mechanism, the flow of blood through choroid arterioles is not independently dependent on changes in intravascular pressure, but instead is influenced by additional blood flow control mechanisms. We found that violet light-induced vasoconstriction was intact in the pressurized whole-eye system; strikingly, increasing intraluminal pressure decreased violet light-induced vasoconstriction (Fig. 2 *F* and *G*). This suggests that violet light promotes myogenic tone in choroid arterioles, and that increasing pressure opposes violet light-induced vasoconstriction, providing a type of autoregulation—but inverted—that enables pressure-dependent control of choroidal blood flow.

### Photomechanical Control of Trans-Retinal Fluid Transport.

**Fig. 3.**
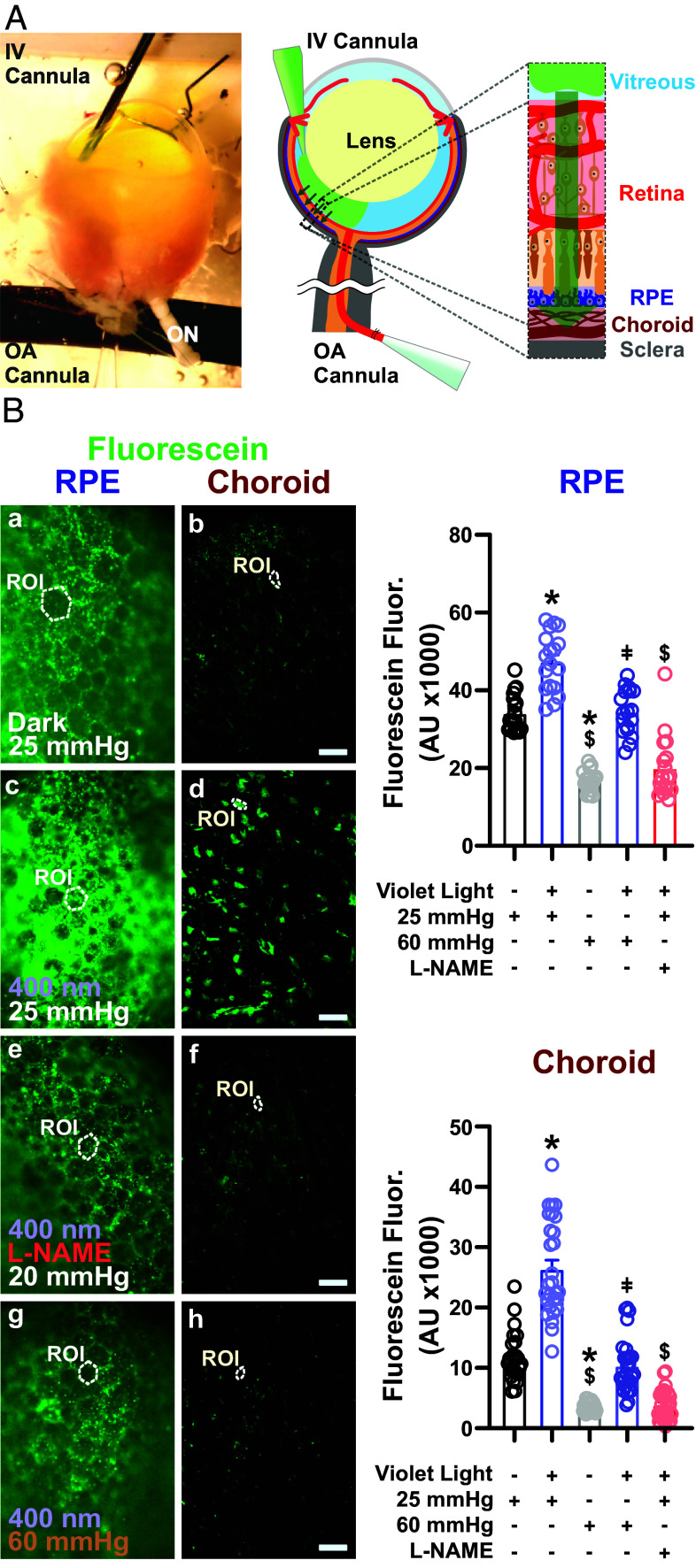
Photomechanical control of subretinal fluid absorption. (*A*, *Left*) Depiction of our ex vivo double-cannulation technique. The enucleated mouse eye is arterially cannulated using a glass pipette, and the vitreous chamber is cannulated using a fine needle for fluorescein injection (3 μM). (Scale bar, 500 µm.) (*A*, *Right*) Schematic illustration of intravitreal injection of sodium fluorescein and its absorption posteriorly into the choroid. (*B*, *Left*) Representative fluorescence micrographs showing fluorescein absorption into the RPE (*Left*) and choroid tissues (*Right*) from the vitreous space under dark conditions at an intraluminal pressure of 25 mmHg (a and b), with violet light stimulation for 60 min at an intraluminal pressure of 25 mmHg (c and d), with violet light stimulation (60 min) and intraluminal L-NAME (10 μM) infusion at 25 mmHg (e and f), and with violet light stimulation (60 min) at 60 mmHg. (Scale bars, 20 μm.) (*B*, *Right*) Summary data showing fluorescein signals in the RPE (*Top*) and choroid (*Bottom*) under the conditions indicated in *B*. Data are presented as means ± SEM (error bars; *P < 0.05 to dark treatment at 25 mmHg; ^$^*P* < 0.05 to violet light treatment at 25 mmHg; and ^ǂ^*P* < 0.05 to dark treatment at 60 mmHg; n = 18 ROIs in 4 choroids from 3 mice per group).

Fluid transport between the photoreceptors, RPE, and choriocapillaris during the visual process serves a number of functions in addition to supplying O_2_ and nutrients to and removing metabolic waste from photoreceptors of the outer retina. These include the removal of heat caused by light absorption, chromophore recycling, K^+^ transport (to maintain photoreceptor excitability), and maintenance of intraocular pressure within the eye. Fluid absorption from the retina into the choroid vasculature, reflecting diffusion of water and solutes across the RPE, relies on the difference between intravascular and intraocular pressure. Changes in retinal illumination lead to transient increases in the extracellular volume (46). Similarly, it has been shown that the transport of fluorescent tracers to the optic nerve following injection into the vitreous is influenced by white light (46, 47). Therefore, we asked whether light-induced effects on the choroid vasculature modulate the trans-retinal transport of retinal fluid into the choroid. For these experiments, we developed a whole-eye, double-cannulated preparation in which one glass pipette is used to cannulate and pressurize the feeding ophthalmic artery, and a second pipette cannula is inserted through the cornea into the vitreous (intravitreous). Following sealing of the cornea to the glass pipette, the intravitreous cannula is pressurized, and sodium fluorescein (376 Da; 2 µg/mL) is injected into the vitreous of the mouse eye (Fig. 3*A*). The whole-eye prep is then pressurized at different intravascular pressures (25 to 60 mmHg) under light and dark conditions for 60 min. The accumulation of sodium fluorescein in RPE–choroid flat mounts is then imaged, and flow from the vitreous to the RPE–choroid complex is examined. Since the RPE is known to play an intermediary role in mass transport from the retina to the choroid, we measured the intensity of sodium fluorescein accumulation from images obtained at both RPE and choroid levels. We found that, in eyes under dark conditions, the absorption of dye into the choroid was attenuated by increased intravascular pressure. Interestingly, this loss in fluid movement was rescued by exposure to violet light (6.1 × 10^14^ photons/cm^2^/s) (Fig. 3 *B* and *C*). Administration of the NOS inhibitor, L-NAME, through the cannula of the ophthalmic artery completely abrogated violet light-induced fluorescein absorption (Fig. 3 *B* and *C*). Collectively, these data suggest that increases in intravascular pressure oppose the vitreous-to-choroid movement of fluid, and that violet light rescues this effect, increasing the movement of fluid into the choroid.

### Expression of OPN3, OPN4, and OPN5 Photopigments in the Choroid Vasculature.

**Fig. 4.**
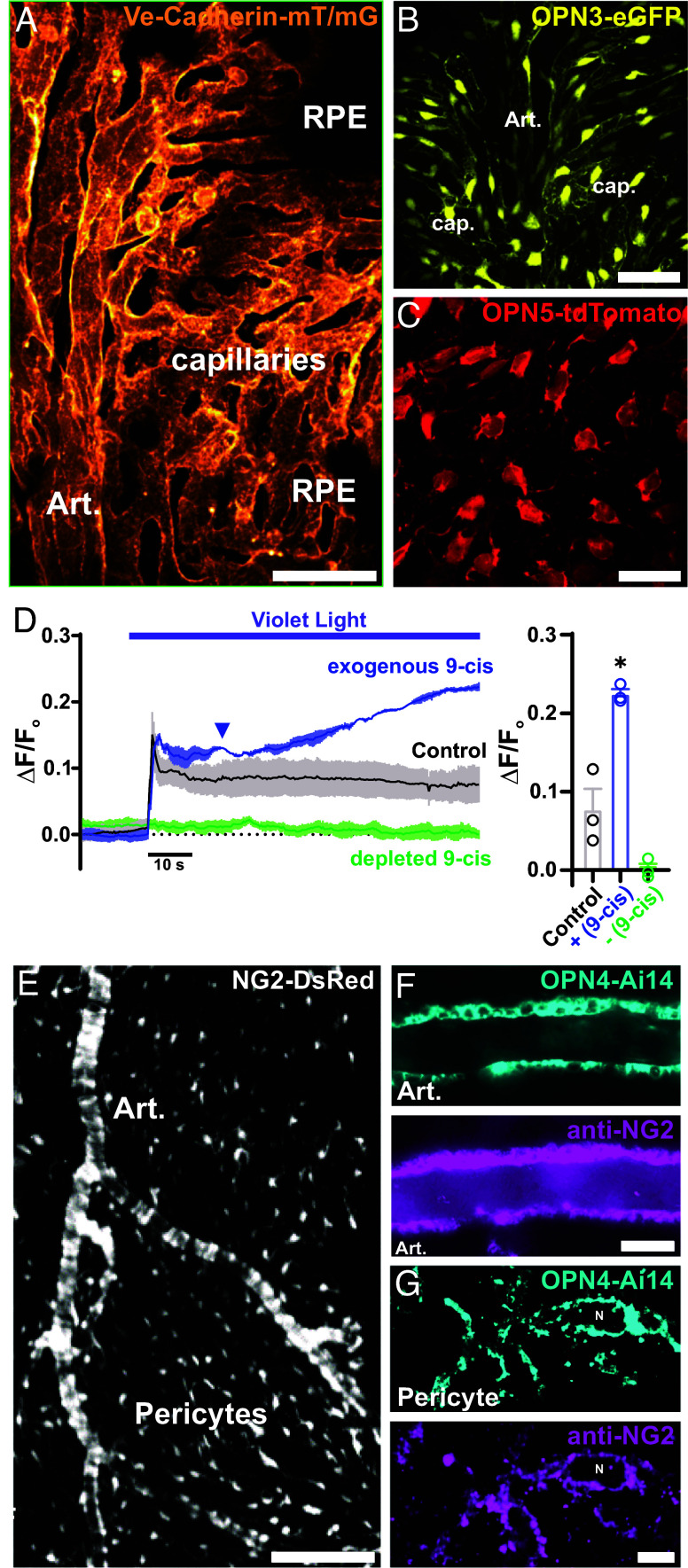
Expression of OPN3, OPN4, and OPN5 photopigments in the choroid vasculature. (*A*) Representative image displaying the ultrastructure of the choriocapillaris endothelium in choroid flat mounts from a Ve-cadherin mT/mG mouse. (*B*) Representative image showing the expression of encephalopsin or panopsin (Opn3) in choroid flat mounts from an Opn3-eGFP mouse. (Scale bar, 50 μm.) (*C*) Representative image showing the expression of neuropsin (Opn5) in choroid flat mounts from an Opn5-tdTomato mouse. (Scale bar, 25 μm.) (*D*) Representative trace and summary data of averaged GcAMP6f fluorescence in the choriocapillaris endothelium in response to constant violet light stimulation (405 nm, 6.1 × 10^14^ photons/cm^2^/s) under control conditions (gray), in the presence of exogenous 9-cis retinal (blue), and following depletion of 9-cis retinal (green). Data are presented as means ± SEM (error bars; *P < 0.05; n = 3 ROIs in 3 choroids from 3 mice per group). (*E*) Representative image showing the zonation of mural cells in choroid flat mounts from an NG2-dsRed mouse. (Scale bar, 40 μm.) (*F* and *G*) Representative images showing melanopsin expression in choroidal SMCs (*F*) and choroidal pericytes (*G*) in choroid flat mounts from an Opn4-Ai14 mouse and immunohistochemical detection of NG2 using an anti-NG2 polyclonal antibody. [Scale bars, 60 μm (*Left*) and 5 µm (*Right*).]

As a step toward elucidating the potential molecular components of the light-sensing apparatus of the mouse choroid, we investigated the cell-specific expression of nonvisual opsins using en face flat-mount choroid preparations from *Opn3*-eGFP, *Opn4*-Ai14, and *Opn5*-Ai14 reporter mice. To provide a landmark for understanding how endothelial cells are organized and structured within the choroidal vasculature, we examined choroids from Vec-mT/mG mice, in which morphological features of Vec-expressing endothelial cells in different regions of the choroid vascular network can be clearly visualized and distinguished (Fig. 4*A*). Arterioles can be identified based on the flattened cobblestone arrangement of their endothelial cells, whereas capillaries are characterized by a tube-like structure formed by individual endothelial cells and the absence of an overlying SMC layer. Choroids isolated from *Opn3*-eGFP and *Opn5-*tdTomato transgenic mice exhibited robust reporter fluorescence in arteriole and choriocapillaris endothelial cells (Fig. 4 *B* and *C*), indicating expression of the corresponding opsins, *Opn3* and *Opn5*. *Opn4*-Ai14 expression was not detected in vascular endothelial cells.

Opsins are light-sensitive owing to the bound chromophore, retinal, which is essential for initiating the light-dependent signaling pathway. Photon absorption leads to 11-cis retinal photoisomerization to all-trans-retinal, which then dissociates from the opsin protein. An exogenous retinal source is not necessary for local opsins, as the RPE–choroid (RPE-Ch) boasts the highest vitamin A concentration in human nonliver tissue (48), potentially even synthesizing retinoic acid (49). However, light-induced photoisomerization and retinal dissociation may deplete local sources. Exogenous application of 9-cis retinal, an aldehyde form of vitamin A (50), enhanced violet light-induced increases in choroid capillary endothelial cell Ca^2+^ (Fig. 4*D*), whereas depleting 9-cis retinal from the tissue through illumination with a 555 nm laser (10% power, 30 to 60 min) (51–53) caused a loss of the violet light response, supporting the idea that violet light stimulates retinal-sensitive opsins, resulting in increased choroid endothelial Ca^2+^ levels.

The structural organization of choroidal mural cells was examined using choroid flat-mounts from NG2DsRedBAC reporter mice expressing DsRed.T1 under the control of the mouse *Cspg4* promoter, which drives NG2 expression. DsRed-driven fluorescence was distributed throughout the choroid vasculature, showcasing the morphology of mural cells (i.e., SMCs and pericytes) (Fig. 4*E*). Within the choriocapillaris, we found a high density—greater than estimated for the retina—of extensively distributed, stellate-shaped, dsRed-positive cells with long, fine processes that extended across and between adjacent vascular lobules in the perivascular space (*SI Appendix*, Fig. S4). As previously proposed (37), because of the high density of dsRed-positivity, we cannot rule out the possibility that other nonvascular cell types within the choroid might also express *Cspg4*. Using opsin-specific reporter mice and immunocytochemistry targeting NG2, we detected *Opn4* and NG2 in arteriolar SMCs (Fig. 4*F*) and choriocapillaris pericytes (Fig. 4*G*). These findings suggest that choroid vascular cells express nonvisual opsins (OPN3, OPN4, and OPN5), potentially constituting an intrinsic light-sensitive, ocular homeostasis-regulating apparatus in the choroid circulation.

## Discussion

With the highest perfusion rate of all vascular beds in the body (54, 55), the choroid vasculature has been the subject of previous studies that have sought to determine whether this unique circulation possesses intrinsic autoregulatory mechanisms that promote vasoconstriction in response to increased pressure (56). Compensatory mechanisms in arterioles—a widespread phenomenon in the body—prevent variations in perfusion pressure from eliciting proportional changes in blood flow. The conventional view has been that, unlike the retina and anterior uvea, the choroid lacks such autoregulatory mechanisms, owing to its high blood flow and low oxygen-extraction rate. Nonetheless, this topic has remained an ongoing debate. In the current study, we directly tested the hypothesis that choroid blood flow is autoregulated; however, not by blood pressure alone, as is the case in traditional myogenic mechanisms, but instead by a dual mechanism involving light sensitivity. Here, we document that the mammalian choroid vasculature is intrinsically sensitive to light, demonstrating that violet light induces constriction of the choroid vasculatures, a feature conferred by the intrinsic sensitivity of endothelial and mural cells comprising the choroid vasculature to the violet-blue spectrum of light. This light sensitivity is key to the operation of an unusual, inverted “myogenic mechanism” in the choroid vasculature: Rather than inducing vasoconstriction, increases in intraluminal pressure act to oppose violet light-induced vasoconstriction, effectively promoting vasodilation. Our findings further demonstrate that the mammalian choroid vasculature is intrinsically sensitive to light, such that violet light induces G_q_PCR signaling-dependent endothelial/mural Ca^2+^-NO signaling, which regulates the movement of trans-retinal fluid. In addition, we propose that this mechanism may be mediated through light activation of OPN3, OPN4, and/or OPN5 in choroid endothelial and mural cells.

### Light-Sensing Pathway in Choriocapillaris Endothelial Cells.

Using transgenic reporter mice for the nonvisual opsins, encephalopsin (*Opn3)* and neuropsin (*Opn5)*, we found that these opsins are expressed in choriocapillaris endothelial cells. We further showed using *Cdh5*-GCaMP6f mice expressing the fast Ca^2+^ indicator, GCaMP6f, specifically in the endothelium that choroid capillary endothelial cells are capable of sensing and rapidly responding to violet light. As part of this process, we propose that violet light stimulates OPN to initiate G_q_PCR-dependent signaling by increasing [Ca^2+^]_i_ through release of Ca^2+^ from the endoplasmic reticulum. OPN3 has been proposed to have both light-dependent (27, 30, 57–59) and -independent functions (60, 61). In contrast, OPN5 and OPN4 initiate Ca^2+^ signaling exclusively in a light-dependent manner. Although Gα_q_-mediated PLCβ activation is quite consistent across cellular contexts, indirect Ca^2+^ elevation by Gα_i_-dependent signaling is variable and even contradictory across systems, as well as within the same cell type. Moreover, synergy between G_i_- and G_q_-dependent signaling has been extensively reported in the literature (62–67). Our results indicate that increased [Ca^2+^]_i_ mediated by G_i_-coupled GPCRs is not only sensitive to pretreatment with the Gα_i_ inhibitor pertussis toxin (PTX), it is also totally abolished by the G_q_-family inhibitor YM-254890. Given the current absence of specific pharmacological inhibitors for different opsins, we suggest that PTX and YM-254890 could be suitable tools for investigating the biological relevance of opsin-coupled Gα_i/o_ and Gα_q_ proteins, respectively.

NO is an important signaling factor in the regulation of choroidal blood flow and microvascular permeability, as supported by numerous animal and human studies (16, 20, 33, 68–72). In the present study, we hypothesized that Ca^2+^-induced NO production is involved in regulating choroidal blood flow during light/dark transitions. Using the fluorescent NO indicator, DAF2-DA, we found that violet-blue light stimulated NO production in choroid capillary endothelial cells, an affect that was abolished by the NOS inhibitor, L-NAME. This finding is consistent with previous studies showing that choroid blood flow/volume decreases in response to stimulation with 473 nm light (11), a decrease that has been hypothesized to reflect light-induced retinal changes (11).

### Light-Sensing Pathway in Choroid Mural Cells.

Using an *Opn4*-Ai14 reporter mouse, we observed that choroid mural cells (vSMCs and pericytes) express melanopsin (OPN4) and respond to light, inducing a VGCC-mediated increase in cytosolic Ca^2+^ and ciliary arteriolar constriction. We hypothesize that light-dependent constriction of feeding arterioles provides an intrinsic mechanism for producing the on-demand reduction in choroidal blood flow that is critical for decreasing the hydrostatic pressure gradient across the choriocapillaris. In so doing, it decreases the resistance to the absorbance of fluid and waste products from the retina during light adaptation. Although light stimulated Ca^2+^ dynamics in capillary pericytes, we did not detect constriction of capillary vessels. Therefore, we hypothesize that Ca^2+^ entering pericytes during the light response may modulate capillary permeability (73) through NO production (74) or matrix metalloproteinase (MMP)-9–mediated degradation of the basement membrane (75, 76). Taken together, our data suggest that violet light-induced ciliary arteriolar constriction, together with increases in pericyte Ca^2+^, fine-tune subretinal fluid absorption at the choriocapillaris during dark/light adaptation. Future studies are warranted to more fully elucidate the detailed mechanisms by which pericytes modulate microvascular fluid absorption in the choroid.

### Vascular Autoregulation of Choroidal Blood Flow.

Myogenic tone is key for limiting blood flow in response to increasing intravascular pressure, but it has been thought that the development of pressure-induced tone is not required for management of flow within the choroid vasculature, reflecting the extremely high perfusion rate within this structure. Using an ex vivo pressurized choroid preparation developed to probe blood flow regulation within the choroid, we found that choroid arterioles and capillaries indeed do not constrict in response to a pressure step (20 to 80 mmHg). However, we found that choroid arterioles, but not the choriocapillaris, develop tone in response to the same pressure step protocol, but only with violet light stimulation. In fact, paradoxically, increasing pressure in the context of violet light stimulation causes vasodilation, opposing violet light stimulation-induced constriction. On the basis of these observations, we hypothesize that, during light adaptation, choroid blood flow decreases, diminishing the hydrostatic pressure of the choriocapillaris and enhancing subretinal fluid absorption. This process serves to eliminate waste and fine-tune O_2_ extraction, which has previously been shown to increase in response to reductions in choriocapillaris flow.

### Violet Light Regulation of Ocular Fluid Homeostasis in Health and Disease.

In mammals, transretinal fluid movement results from pressure gradients caused by high plasma protein concentrations in the choroid (oncotic) and greater interstitial fluid pressures in the retina (hydrostatic). Light-dependent reductions in choroidal blood flow, and attendant decreases in oncotic and hydrostatic pressure, may facilitate fluid reabsorption into choroid capillaries. In myopia, also known as nearsightedness, light is focused incorrectly in front of the retina instead of directly on it owing to a refractive error, causing distant objects to appear blurry while allowing close objects to be seen clearly. Despite the growing worldwide occurrence of myopia, with estimates indicating that nearly half (49.8%) of the global population will be affected by 2050 (15), the exact causes and mechanisms that trigger the onset of this condition remain poorly understood. Although the role of the choroid in myopia is still being investigated, research suggests that alterations in choroidal thickness and positioning of the retina may play a role in the pathogenesis of the condition (77). Myopic eyes exhibit reduced choroidal blood flow (for review see ref. 78). Studies in mice, chicks, and humans indicate that visible violet light protects against myopia development (79) and the absence of violet light in modern society is proposed as a potential cause of the surge in myopia cases (80), therefore changes in the modern lighting environment are considered a contributing factor (79, 80). Importantly, we show that violet light can directly constrict feeding choroidal arterioles, resulting in a decrease in blood flow. Therefore, light-sensitive mechanisms that regulate choroid blood flow and ocular fluid homeostasis may play a crucial role in ocular pathologies, including myopia. Previous studies suggest that OPN3 (81) and OPN5 (80) are involved in the development of myopia. Therefore, pharmacological targeting of this light-sensitive mechanism could mitigate myopia progression and dry age-related macular degeneration pathogenesis.

## Methods

All animal studies were performed in accordance with guidelines of the Institutional Animal Care and Use Committee (IACUC Approval No. 20-07-1039-1) of the University of Nevada, Reno; University of Washington, School of Medicine, Seattle; and University of Cincinnati, College of Medicine. The following mice strains were used C57BL/6J (Stock No. 000604; Jackson Laboratories); NG2-DSRed (stock no. 008241); the previously described *Cdh5*-GCaMP6f transgenic mouse line (40), in which the high signal-to-noise Ca^2+^ indicator GCaMP6f is expressed under the transcriptional control of the *Cdh5* promoter; and NG2-GCaMP6f mice, generated by crossing NG2_Cre_ mice (008533; Jackson Laboratory, Bar Harbor, ME) with floxed GCaMP6 mice (024106; Jackson Laboratory). Eyes from *Opn3*-eGFP, *Opn4*-Ai14, and *Opn5*-tdTomato reporter mice were received from the University of Washington, School of Medicine, Seattle, and the University of Cincinnati, School of Medicine The ex vivo whole-eye model, developed by our group, was prepared as previously described (31). The choroid vasculature was photostimulated using a Mightex Polygon DMD pattern illuminator, which provides precise spatiotemporal, high-resolution control of light delivery, and a dedicated 7-wavelength (405, 445, 470, 520, 528, 555, and 640 nm) laser launch (LDI-7, 89 North). For dark/light treatment experiments, the choroid vasculature was pressurized for 60 min at different perfusion pressures (25 to 60 mmHg) using a Mightex photostimulation system (Polygon1000-G). Data in figures and text are presented as means ± SEM unless otherwise stated. Expanded methods are provided in *SI Appendix*.

## Supplementary Material

Appendix 01 (PDF)

## Data Availability

High-speed, high resolution spinning disk confocal/widefield imaging obtained from ex vivo pressurized and unpressurized choroid preparations have been deposited in Dryad data platform (https://datadryad.org/stash) (82). Images in TIFF format will be generated using commercial software (VisiView), but images and metadata are freely accessible using open-source software such as ImageJ. Immunofluorescence/brightfield microscopy images generated from pinned-down en face retinal vasculatures. Raw z-stack images will be saved in either TIFF or AVI format and can be freely transformed within open-source software.
