# Supplementary material for: Light-sensitive Ca2+ signaling in the mammalian choroid
Source: Proc Natl Acad Sci U S A. 2024 Nov 8;121(46):e2418429121. doi: 10.1073/pnas.2418429121 (PMC11573543; doi:10.1073/pnas.2418429121)
Supplement: Supplementary file 1 — Appendix 01 (PDF) [file pnas.2418429121.sapp.pdf]

Supplement Information for

Light Sensitive Ca^{2+} Signaling in the Mammalian Choroid.

Ahmed M. Eltanahy[1], Alex Aupetit[1], Ethan D. Buhr[2,5], Russell N. Van Gelder[2,3,4,5], and Albert L. Gonzales[1]

[1]Department of Physiology and Cell Biology, University of Nevada School of Medicine, Reno, NV, 89557-0318, USA

[2]Departments of [2]Ophthalmology, [3]Laboratory Medicine and Pathology, and [4]Biological Structure, and [5]Roger and Angie Karalis Retina Center, University of Washington, Seattle, WA, 98104, USA

Corresponding Author: Albert L. Gonzales

Email: albertgonzales@unr.edu

Methods

Animal models

All animal studies were performed in accordance with guidelines of the Institutional Animal Care and Use Committee (IACUC approval no. 20-07-1039-1) of the University of Nevada, Reno; University of Washington, School of Medicine, Seattle; and University of Cincinnati, College of Medicine. Adult (2–5-month-old) male and female mice were group-housed on a 12-hour light:dark cycle with environmental enrichment and free access to food and water. Animals were euthanized by 4% isoflurane anesthesia followed by rapid decapitation. All experimental protocols used in this study are in accord with institutional guidelines approved by the Institutional Animal Care and Use Committee of the University of Nevada Reno. The following mice strains were used: C57BL/6J (stock no. 000604; Jackson Laboratories); NG2-DSRed (stock no. 008241); the previously described *Cdh5*-GCaMP6f transgenic mouse line (40), in which the high signal-to-noise Ca^{2+} indicator GCaMP6f is expressed under the transcriptional control of the *Cdh5* promoter; and NG2-GCaMP6f mice, generated by crossing $NG2_{Cre}$ mice (008533; Jackson Laboratory, Bar Harbor, ME) with floxed GCaMP6 mice (024106; Jackson Laboratory). Eyes from *Opn3*-eGFP, *Opn4*-Ai14, and *Opn5*-Ai14 reporter mice were received from the University of Washington, School of Medicine, Seattle, and the University of Cincinnati, School of Medicine. The Opn3-eGFP reporter mouse is a genetically modified Opn3-eGFP (enhanced green fluorescent protein) reporter mouse strain that harbors an eGFP reporter gene inserted at the start of exon 1 of the coding sequence of Opn3. Because this is positioned downstream of the Opn3 promoter, eGFP expression is driven by the Opn3 promoter. To assess the expression of Opn4 and Opn5 in different choroidal cell types, we used Opn4cre/+;Ai14 (R26-LSL-tdTomato) and Opn5cre/+;Ai14 (R26-LSL-tdTomato) mice, in which tdTomato is expressed in the Opn4 or Opn5 lineage, respectively. *NG2DsRedBAC* reporter mice [*Tg(Cspg4-DsRed.T1)1Akik/J*] express the red fluorescent protein *DsRed.T1* under the control of the mouse *Cspg4* (chondroitin sulfate proteoglycan 4) promoter, which drives expression of the mural cell marker, NG2 (neural/glial antigen 2).

Cynomolgus monkeys were housed and maintained at Charles River Laboratories, Preclinical Services (Reno, NV, USA). The Institutional Animal Care and Use Committee (IACUC approval no. I-000536, Charles River Laboratories). *Cynomolgus monkeys* of either sex (2–8 years of age) were sedated with ketamine HCL (10 mg kg^{-1} given intramuscularly; Zoetis, Parsippany, NJ, USA) and euthanized using pentobarbital (I.V., Fatal-Plus; Vortech Pharmaceuticals, Ltd, Dearborn, MI, USA) and exsanguination. The dosage for pentobarbital was 0–4.5 kg, 1.0 ml; 4.6–9.1 kg, 2.0 ml and >9.2 kg, 3.0 ml. Tissue samples from these animals were provided immediately after necropsy to the laboratories within the Department of Physiology and Cell Biology at the University of Nevada, Reno. Freshly isolated monkey eyes were dissected immediately at the facilities of the University of Nevada, Reno, and used for *ex vivo* experiments and imaging. All non-human primate tissues used in the present study came from animals that had been used previously for pharmaceutical testing, and no non-human primates were purchased, bred, or killed specifically for the current study.

Solutions and reagents

Retina dissection buffer consists of 119 mM NaCl, 3 mM KCl, 0 mM $CaCl_2$, 3 mM $MgCl_2$, 5 mM glucose, 26.2 mM $NaHCO_3$ and 1 mM NaH_2PO_4, bubbled with 95% O_2/5% CO_2 (pH 7.4). Retinal physiological saline solution (PSS; pH 7.4) contains 124 mM NaCl, 26 mM $NaHCO_3$, 1 mM NaH_2PO_4, 2.5 mM KCl, 1.8 mM $CaCl_2$, 2 mM $MgCl_2$ and 10 mM glucose, bubbled with 95% O_2/5% CO_2 (31, 41). Ca^{2+}-free and Mg^{2+}-supplemented PSS contains 124 mM NaCl, 26 mM

$NaHCO_3$, 1 mM NaH_2PO_4, 2.5 mM KCl, 3.8 mM $MgCl_2$ and 10 mM glucose, supplemented with 6 μM cis-retinal. All chemicals and drugs were purchased from Cayman Chemical (USA) and Sigma-Aldrich (St. Louis, MO, USA) unless specified otherwise.

Immunohistochemistry

Following euthanasia, eyes were enucleated and placed in 4% paraformaldehyde, diluted in phosphate-buffered saline (PBS), for 2 hours at 4°C. Whole retinas were dissected from eyes, blocked, and permeabilized by incubating with PBS containing 5% bovine serum albumin (BSA) and 0.2% Triton X-100 at room temperature for 2 hours. Choroids were then incubated for 24 hours at 4°C with primary antibody, diluted in PBS containing 0.5% BSA and 0.2% Triton X-100. The following conjugated antibodies/stains were used at the indicated dilutions: anti-NG2 antibody 1:1000 (ab129051). Choroids were washed three times in PBS for 10 minutes each at room temperature, after which radial slits were made to create a flat-mount preparation, which was then placed under coverslips and stabilized using Vectashield Plus mounting media (Vector Laboratories). Images were acquired with an upright 10X air (0.1 NA) or 20–60X water-immersion objective utilizing widefield microscopy.

Pressurized and non-pressurized ex vivo choroid preparation

The *ex vivo* whole-eye model, developed by our group, was prepared as previously described (31). Briefly, after euthanizing mice, the overlying skull and brain were carefully removed, after which the eye, surrounding orbit bones, connected musculature, vasculature, and optic nerve were removed in one piece using angled eye scissors and placed in 4°C Ca^{2+}-free retina dissection buffer. The surrounding bones and outer muscles were removed into a dissection dish at 4°C with intermittent exchange of bubbled dissection solution. The inner muscles and connective tissue were then removed, revealing the ophthalmic artery, which enters the retina at the base of the optic nerve. The cornea was pierced using fine forceps, and the entire cornea and iris were removed by making a circumferential cut. The lens was grasped with fine forceps, after which the connective tissue of the ora searrata was disrupted and the lens was removed using closed, semi-fine, blunt forceps. Fine dissection scissors were used to remove the retina, leaving the RPE-choroid-sclera complex intact. For the non-pressurized choroid preparation, the RPE-choroid-sclera complex was flat-mounted and imaged using a confocal microscope while superfusing the tissue with retina physiological saline solution. The RPE layer can be removed by gently flushing of bath solution buffer using a micropipette. For the pressurized preparation, the dissected choroid was first transferred to a custom myography chamber containing room temperature retina dissection buffer using a large barrel transfer pipette. The ophthalmic artery was then cannulated using a pulled borosilicate glass pipette (~100–150 μm tip diameter) filled with retina PSS, maintained at pH 7.4 by bubbling with 95% O_2/5% CO_2 and supplemented with 6 μM cis-retinal. Loose ophthalmic artery branches were tied off using a monofilament nylon fiber (cat. THR-G; Living Systems), and the cannulated vessel was secured to the pipette.

The tip of the cannulation pipette was advanced in such a manner that the downstream ophthalmic artery was narrower than the tip of the pipette, a maneuver that allows the remaining ophthalmic artery and retinal artery vasculature, rather than the tip of the pipette, to set inlet flow. The choroid was placed over a custom silicone platform and flattened by making radial cuts in the outer edges, after which the choroid was secured to the platform using short pins cut from a 70-μm diameter tungsten wire. The cannulation pipette was attached to a pressure transducer to allow measurement of pressure at the level of the ophthalmic artery and connected to two solution columns, linked via a 3-way adjustable valve. One column was fixed at a height providing a pressure of 25 mmHg and the other was adjustable, providing a full range of pressures from 0 to 100 mmHg. This allowed for sustained pressure steps by incrementally changing the adjustable

column height. Successful choroid vessel cannulation was confirmed by pressurizing the choroid to 25 mmHg and monitoring the rapid elimination of blood cells from the vasculature via brightfield microscopy. Bath perfusion (with bubbled PSS) was kept constant at 5 mL/min (3 mL/min for microelectrode experiments), and a temperature of 35–36°C was maintained using an inline heater. The preparation was allowed to equilibrate for 20 minutes at 25 mmHg before the start of experimentation.

For the double-eye cannulation technique, we used a 35-gauge stainless steel needle connected to polyethylene tubing and a 20 μL Hamilton syringe. The tubing was first loaded with 5 μL of Na^+-fluorescein (Sigma), after which the needle was placed in the vitreous using a trans-corneal approach so as to avoid puncturing the choroid circulation, and 3 μL of fluorescein was injected at a rate of 1 μL/min using a syringe pump (PHD ULTRA; Harvard Apparatus). After perfusing the whole eye for 1-hour, trans-retinal fluorescein absorption was quantified at the level of the RPE and choriocapillaris endothelium by flat mounting and confocal microscopy imaging. Mean fluorescence intensity values of pixels for RPE and choroid endothelial cells were quantified using ImageJ.

Light stimulation

The choroid vasculature was photostimulated using a Mightex Polygon DMD pattern illuminator, which provides precise spatiotemporal, high-resolution control of light delivery, and a dedicated 7-wavelength (405, 445, 470, 520, 528, 555, and 640 nm) laser launch (LDI-7, 89 North). For dark/light treatment experiments, the choroid vasculature was pressurized for 60 minutes at different perfusion pressures (25–60 mmHg) using a Mightex photostimulation system (Polygon1000-G).

Ca^{2+}, NOS, Fluo4-AM and calcein-AM imaging

Changes in endothelial cell and mural cell Ca^{2+} levels were assessed by monitoring changes in cytosolic Ca^{2+} concentration, measured as changes in GCaMP6f fluorescence intensity over time. GCaMP6f fluorescence was imaged using a stand-alone Crest Optics X-Light V3 spinning-disk confocal and widefield microscope with a dedicated, 7-wavelength (405, 445, 470, 520, 528, 555, and 640 nm) laser launch (LDI-7; 89 North) and dual low-light ORCA-Fusion Gen-III sCMOS Cameras (Hamamatsu) attached to an Olympus BX51WI microscope base. GCaMP6f fluorescence was excited using a 488 nm solid-state laser, and the emitted fluorescence was collected through a 520-nm band-pass filter. Images were acquired at a rate of 0.5 frames/s with an exposure time of 200 ms at 0.1% laser power, parameters that minimize cross-stimulation by imaging light. Because of the sensitivity of the cMOS cameras, these acquisition settings were sufficient to obtain fluorescent images without stimulating the tissue. The parameter, $\Delta F/F_0$, for an intensiometric indicator is determined as the fluorescence intensity change registered during the change in Ca^{2+} (ΔF) divided by the average baseline fluorescence (F_0) under resting conditions. NO production was measured by incubating flat-mounted choroids with 1 μM DAF-2 for 30 minutes and then measuring the change in fluorescence of choroid endothelial cells at 37°C by confocal microscopy using excitation and emission wavelengths of 488 nm and 520 nm, as previously reported (83). Monkey tissues were visualized by loading flat-mounted monkey choriocapillaris patches with either 1 μM calcein-AM or 10 μM Fluo-4 am. In the latter case, 50 μg Fluo-4-AM and 1% pluronic acid-127 (Molecular Probes), dissolved in a total volume of 50 μL DMSO, were loaded into the tissue by bath application (final Fluo-4-AM concentration, 10 μM).

Statistical analysis

Data in figures and text are presented as means ± standard error of the mean (SEM) unless otherwise stated. All experiments were performed in a randomized manner (animals, pharmacological treatments). Statistical tests were performed using GraphPad Prism 9 software. Statistical significance was determined using paired Student's t-tests and one-way analysis of variance (ANOVA). P-values ≤ 0.05 were considered statistically significant for all experiments.

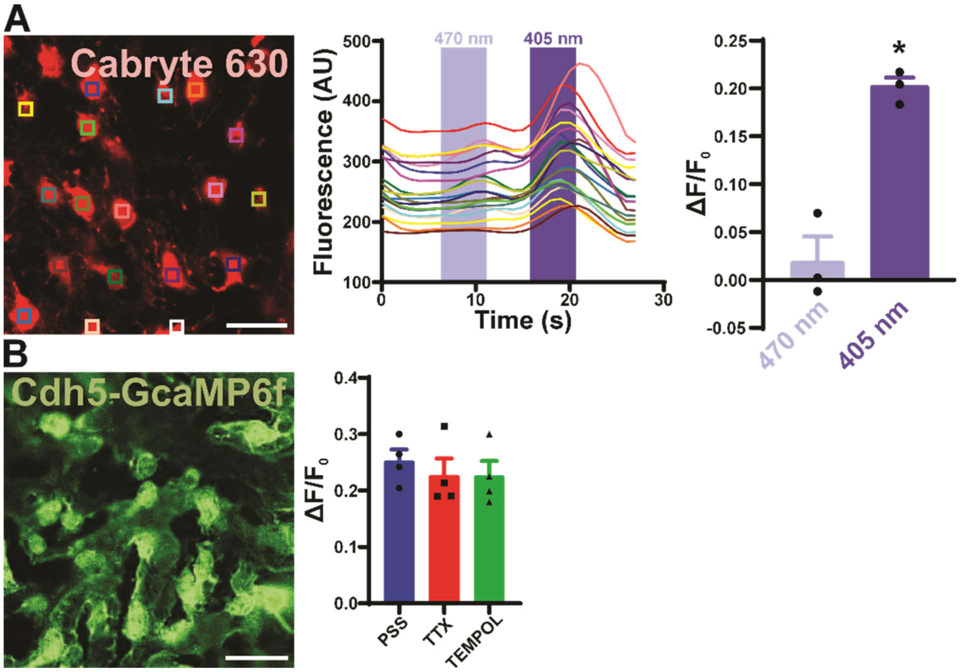

Supplement Figure 1: ***Violet light Stimulate Vasculature Ca^{2+} Signals.*** **A:** Representative image, traces and summary data of Cabryte 630–loaded choriocapillaris following light stimulation at 470 nm (*6.1 × 10^{14}* photons/cm^2/s) and 405 nm (*6.1 × 10^{14}* photons/cm^2/s) (n = 3 choroid preparations) Scale bars: 30 μm *P < 0.05 to 470 nm **B:** Representative image and summary data showing changes in choriocapillaris endothelial Ca^{2+} signaling using *Cdh5*-GCaMP6f transgenic mice in response to violet light stimulation under control conditions (PSS) and following treatment with Tetrodotoxin (TTX, 500 nM) or TEMPOL (4-hydroxy-2,2,6,6-tetramethyl-1-piperidinyloxy, 10 μM) (n = 4 choroid preparations) Scale bars: 30 μm.

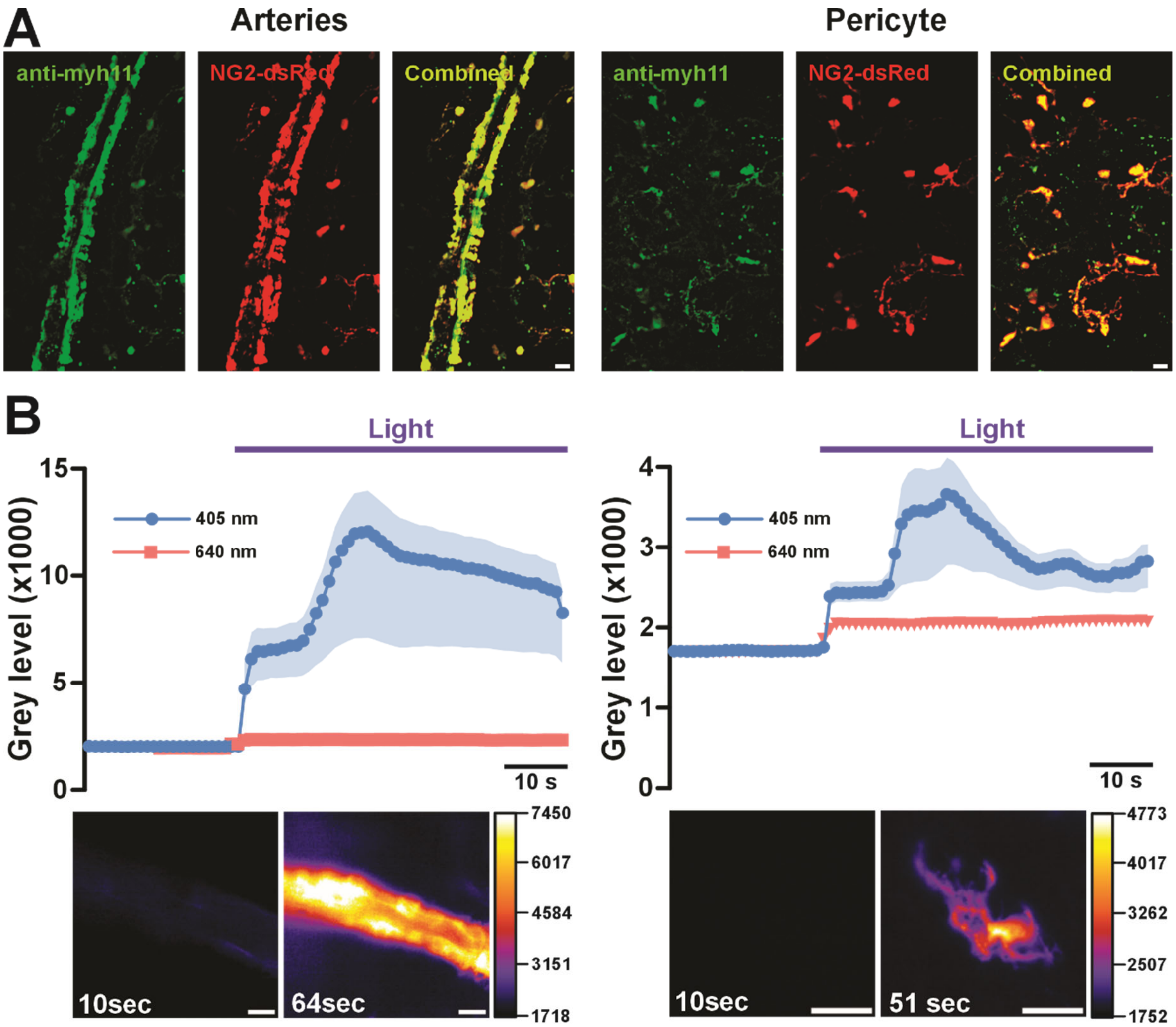

Supplemental Figure 2: ***Violet light stimulates Ca^{2+} and Constriction in Choroids isolated from Myh11-GCaMP6f Transgenic Mice.*** **A**: Representative images showing melanopsin expression in choroidal arterioles (left) and choroidal pericytes (right) in choroid flat mounts from an NG2-dsRed mice (red) and immunohistochemical detection of MYH11 using an anti-myh11 polyclonal antibody (green). Scale bar = 10 μm. **B**: Representative traces (top) and images (bottom) of a Myh11-GCaMP6f-Ai95D mouse choroidal preparation showing changes in fluorescence in response to light stimulation (405 and 640 nm) or arterioles (left) and pericytes (rgith). Scale bar =10 μm

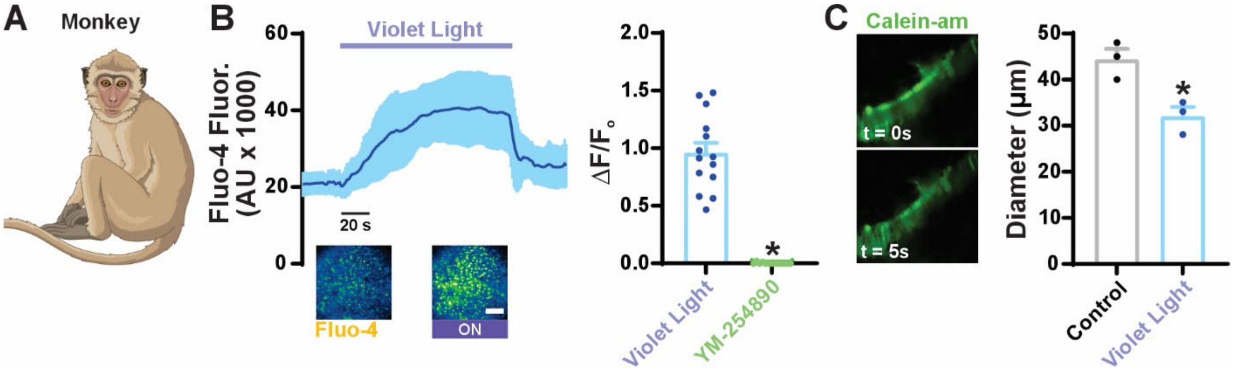

Supplement Figure 3. ***Intrinsic Light-Sensitive Ca^{2+} Signaling in the Primate Choriocapillaris Endothelium.*** **A:** Schematic illustration of an *ex vivo* choroid preparation from freshly isolated *Cynomolgus* monkey eyes. **B (top left):** Representative average fluorescence trace from Fluo-4-AM–loaded choriocapillaris endothelium following violet light stimulation (405 nm, 6.1×10^{14} photons/cm^2/s) (n = 3 choroid preparations from 3 monkeys). **B (bottom left):** Representative micrographs of a monkey choroidal preparation showing changes in Fluo-4 fluorescence in response to violet light stimulation (405 nm). Scale bars: 50 µm. **B (right):** Summary data showing changes in Fluo-4 fluorescence in the choriocapillaris endothelium in response to violet stimulation (405 nm, 6.1×10^{14} photons/cm^2/s) under control conditions and in the presence of YM-254890 (1 µM). Data are presented as means ± SEM (error bars; *$P < 0.05$ to violet light; n= 8 regions of interest (ROIs) in 3 choroids from 3 monkeys per group). **C)** Representative images (**left**) and arteriolar diameter in response to stimulation with violet light (405 nm, 6.1×10^{14} photons/cm^2/s). Data are presented as means ± SEM (error bars; *$P < 0.05$ to control; n = 3 vessels in 3 choroids from 3 monkeys per group). Representative micrographs of calcein-AM staining of the choroid vasculature before and 5 seconds after stimulation with violet light. Scale bars = 20 µm

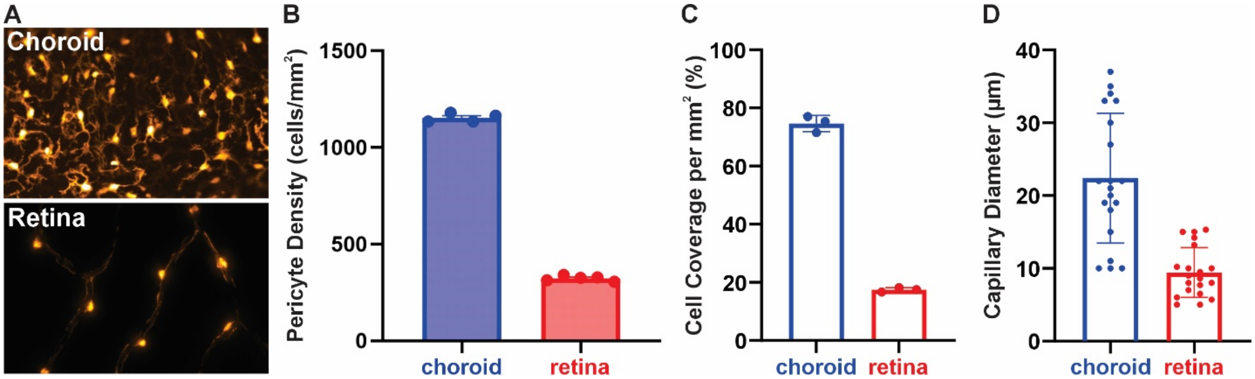

Supplement Figure 4: ***Choroid Express Greater Number of Capillary Pericytes*** **A:** Representative images of the choroid and retina of NG2dsRed mice. **B:** Summary data showing average quantification of pericyte density (cells/mm^2) in the choroid and retina n=4 retinas/choroids from 4 mice. *$P < 0.05$. **C:** Percentage of cell coverage / mm^2 (n=3 for each group from 3 mice, *$P < 0.05$). **D:** Summary data showing capillary diameters in the choroids and retinas from NG2dsRed mice in µm. *$P < 0.05$**.**
